# Resistance to chemotherapy: new treatments and novel insights into an old problem

**DOI:** 10.1038/sj.bjc.6604510

**Published:** 2008-07-29

**Authors:** S Raguz, E Yagüe

**Affiliations:** 1 MRC Clinical Sciences Centre, Imperial College Faculty of Medicine, Hammersmith Hospital, Du Cane Road, London W12 0NN, UK; 2 Division of Surgery, Oncology, Reproductive Biology and Anaesthetics, Department of Oncology, Imperial College Faculty of Medicine, Hammersmith Hospital, Du Cane Road, London W12 0NN, UK

**Keywords:** drug resistance, chemotherapy response, cancer stem cells, genome profiling, chronic myelogenous leukaemia, breast cancer

## Abstract

Resistance to cancer chemotherapeutic treatment is a common phenomenon, especially in progressive disease. The generation of cellular models of drug resistance has been pivotal in unravelling the main effectors of resistance to traditional chemotherapy at the molecular level (i.e. intracellular drug inactivation, detoxifying systems, defects in DNA repair, apoptosis evasion, membrane transporters and cell adhesion). The development of targeted therapies has also been followed by resistance, reminiscent of an evolutionary arms race, as exemplified by imatinib and other BCR-ABL inhibitors for the treatment of chronic myelogenous leukaemia. Although traditionally associated with the last stages of the disease, recent findings with minimally transformed pretumorigenic primary human cells indicate that the ability to generate drug resistance arises early during the tumorigenic process, before the full transformation. Novel technologies, such as genome profiling, have in certain cases predicted the outcome of chemotherapy and undoubtedly have tremendous potential for the future. In addition, the novel cancer stem cell paradigm raises the prospect of cell-targeted therapies instead of treatment directed against the whole tumour.

Gilman and co-workers were the first to introduce chemotherapy into clinical practice at the end of the Second World War when they used nitrogen mustard to treat a patient with advanced malignant lymphoma. After an initial regression of the disease, a second course of therapy was given at a reduced dose due to the toxicity of the treatment, with an associated lesser therapeutic effect. By the time the third treatment was given, the tumour no longer responded to the chemotherapeutic agent (Goodman *et al*, 1946). Since then, chemotherapy has been one of the main therapeutic strategies in cancer treatment, but, to paraphrase Paul Ehrlich, resistance has followed as a faithful shadow.

Chemotherapeutic strategies have used a variety of drugs and hormonal agents that interfere with the basic machinery of the cell. Subsequent improved understanding of the molecular alterations present in the cancer cell has enabled the development of targeted therapies for some forms of cancer. Interestingly, resistance appears not only to traditional chemotherapy but also to targeted therapies such as tamoxifen, which targets the oestrogen receptor (ER) in breast cancer (Ali and Coombes, 2002); imatinib, which targets the kinase activity of the translocated *BCR-ABL* in chronic myelogenous leukaemia (CML) (Weisberg *et al*, 2007); or gefitinib, which inhibits epidermal growth factor receptor (EGFR) kinase (Engelman *et al*, 2007).

## Mechanisms of resistance to traditional chemotherapy

Two main groups of factors contribute to the development of drug resistance. The first group includes pharmacological and physiological factors such as drug metabolism and excretion, inadequate access of the drug to the tumour, inadequate infusion rate and inadequate route of delivery. These are extremely important issues not only in clinical practice but also fundamental in drug development (Garattini, 2007). The second group includes cell- or tissue-specific factors. The cytochrome *P*450 enzymes, a multigene family of constitutive and inducible haem-containing oxidative enzymes from the liver, play an important role in the metabolism of a diverse range of xenobiotics and are often overexpressed in a variety of solid tumours in which they can contribute to drug resistance. Drug analogues of DNA precursors such as 5-fluorouracil and cytosine arabinoside require metabolic activation, and resistance can arise from modification of these activation pathways. Altered topoisomerase I and II activity prevents drugs such as doxorubicin, etoposide and camptothecin from binding the topoisomerase–DNA complex, allowing the broken strands to be repaired. Many anticancer drugs, such as platinum compounds, alkylating agents and nitrosoureas, cause direct damage to the structural integrity of the DNA, and resistance to these compounds results from activation of DNA repair systems. Regulation of cell death by evasion of apoptosis, necrosis, mitotic catastrophe (Mansilla *et al*, 2006) or evasion of senescence (Dimri, 2005) contributes towards drug resistance. In addition, the differential expression of membrane proteins such as solute carriers, channels and ATP-binding cassette (ABC) transporters (Gottesman *et al*, 2002; Huang *et al*, 2004) have all been demonstrated to play an important role in drug resistance. Although these mechanisms have been clearly demonstrated in cell culture, their relevance with the clinical outcome of chemotherapy is less clear (Cimoli *et al*, 2004; Uggla *et al*, 2007) and has only been demonstrated for some of these mechanisms (Clarke *et al*, 2005).

## Molecular mechanisms of resistance to targeted chemotherapy

### CML and imatinib

Chronic myelogenous leukaemia was the first human cancer to be associated with a consistent chromosomal abnormality, the Philadelphia chromosome, a translocation that juxtaposes the 3′ sequence from the *ABL1* proto-oncogene on chromosome 9 with the 5′ sequence from the *BCR* gene on chromosome 22. The resultant chimaeric BCR-ABL protein is a constitutively active protein tyrosine kinase with an important role in the regulation of cell growth (Melo and Barnes, 2007). Traditional therapy for CML includes initial allogenic stem-cell transplantation and interferon-*α*, followed by second-line treatment with hydroxyurea or busulfan in non-responsive patients. Imatinib mesylate (formerly STI571; Gleevec, Novartis, Basel, Switzerland) is a potent and highly specific competitive inhibitor of the BCR-ABL tyrosine kinase. Initially, it had a high rate of cytogenetic and haematologic responses in patients with chronic-phase CML in whom previous therapy had failed, and its use has revolutionised the management and clinical expectations of CML patients. Unfortunately, not long after its initial use, resistance to imatinib was demonstrated in CML patients (Gorre *et al*, 2001). Approximately 50% of imatinib-resistant CML patients carry a resistance-associated point mutation in *BCR-ABL*, which interferes with imatinib binding. More than 50 different resistance-associated point mutations have been described to date. These findings have spurred the development of second-generation BCR-ABL inhibitors such as BMS-354825, which has a two-log increased potency relative to imatinib and retains its inhibitory activity against 14 of 15 imatinib-resistant BCR-ABL mutants tested (Shah *et al*, 2004). However, resistance to some of these second-generation inhibitors, such as nilotinib and dasatinib, has already been described. This indicates that the potential for new drug-resistant point mutations in BCR-ABL persists and justifies the continued development of more potent BCR-ABL inhibitors (Weisberg *et al*, 2007). In the remaining 50% of imatinib-resistant patients with no *BCR-ABL* mutations, *BCR-ABL* gene amplification or overexpression at the mRNA and protein levels has been detected in clinical samples (Hochhaus *et al*, 2002). In addition, chromosomal aberrations, reduced intracellular uptake of imatinib and the disease phase have all been implicated in imatinib resistance (Nimmanapalli and Bhalla, 2002).

### Breast cancer, tamoxifen, aromatase inhibitors and trastuzumab

Breast cancer accounts for one in four of all female cancers, making it by far the most common cancer in women in the western world, where one in nine women will develop the disease at some stage in their lives. Breast cancer treatment involves surgical removal of the tumour, although this is ineffective if malignant cells have escaped from the site of the primary tumour. Discovery of the involvement of the ovarian hormone oestrogen and its mechanism of action (Dickson and Lippman, 1995) paved the way for the development of therapies for ER-positive patients that inhibit oestrogen action. These therapies include tamoxifen, which blocks the ER, and the oestrogen synthetase (aromatase) inhibitors formestane and exemestane, which inhibit oestrogen synthesis (Ali and Coombes, 2002). Despite the huge improvement in cancer survival due to tamoxifen treatment, some patients relapse and the use of sequential therapy with exemestane after 2–3 years of tamoxifen treatment has improved disease-free survival, as compared with the standard 5 years of tamoxifen treatment alone (Coombes *et al*, 2004). The use of endocrine agents has markedly reduced the number of deaths from breast cancer over the past decades. However, in many cases, these therapies fail due to recurrent endocrine-resistant tumours, and much effort is being made to elucidate the mechanisms that underlie resistance to endocrine therapies (Weinberg *et al*, 2005). Altered growth factor signalling, notably EGFR (Schiff *et al*, 2005) and insulin-like growth factor I receptor (IGF-IR) make a significant contribution to the development of antioestrogen resistance, and these have been reviewed recently (Baselga, 2006).

Up to 25% of patients diagnosed with breast cancer have tumours that overexpress the EGFR-2 (HER2 or Erb B-2). HER2-positive breast cancer is highly proliferative, difficult to treat and confers a poor prognosis. Trastuzumab is a monoclonal antibody targeted against the HER2 tyrosine kinase receptor. The majority of patients with metastatic breast cancer, who initially respond to trastuzumab, develop resistance within 1 year of treatment initiation, and in the adjuvant setting, 15% of patients still relapse despite trastuzumab-based therapy. Preclinical studies have indicated several molecular mechanisms that could contribute to the development of trastuzumab resistance. One major determinant to resistance is increased signalling via the phosphatidylinositol 3-kinase/Akt pathway. This results in the activation of multiple receptor pathways, including HER2-related receptors and non-HER receptors such as the IGF-IR, which appear to be involved in a cross talk with HER2 in resistant cells (Berns *et al*, 2007). Alternatively, the loss of function of the tumour suppressor PTEN, the negative regulator of Akt, results in an increase in Akt signalling that leads to decreased trastuzumab sensitivity. Decreased interaction between trastuzumab and its target receptor HER2, which is due to steric hindrance of HER2 by cell-surface proteins such as mucin-4 (MUC4), can block the inhibitory actions of trastuzumab. Novel therapies targeted against these aberrant molecular pathways offer hope that the effectiveness and duration of response to trastuzumab can be greatly improved (Baselga, 2006; Nahta *et al*, 2006). As only about one-third of breast cancer patients overexpressing HER2 respond to trastuzumab monotherapy, the identification of predictive biomarkers that can more accurately select responders or non-responders is vital, not only to improve its therapeutic index, but also to gain insight into the molecular pathways involved in trastuzumab resistance and to rationally design successful combination therapies.

## The ability to acquire drug resistance arises early during the tumorigenesis process

**Figure 1:**
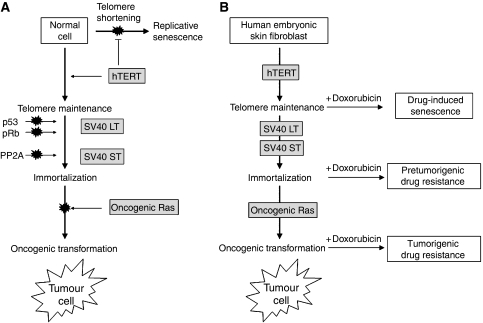
Cellular model of tumorigenesis and pretumorigenic drug resistance. (**A**) This model was first described by Hahn *et al* (1999) in human BJ fibroblasts and embryonic kidney epithelial cells. Normal cells are transformed by expressing the catalytic subunit of telomerase (to avoid replicative senescence), SV40 LT (which binds and inactivates the tumour suppressors p53 and pRb controlling the DNA repair and G1 cell-cycle checkpoints, respectively) and ST (which binds and inactivates PP2A, a serine/threonine phosphatase involved in several signalling pathways) proteins and oncogenic ras. Since then, the model has been validated by transforming primary epithelial cells from breast, prostate, ovary and lung (Boehm and Hahn, 2005). (**B**) Complete tumorigenic transformation is not a prerequisite for the acquisition of drug resistance. When a series of pretumorigenic and minimally transformed tumorigenic cells derived from human embryonic skin fibroblasts are treated with doxorubicin, drug-resistant cells can be obtained from fully tumorigenic as well as pretumorigenic cells. Cells at the early stages of transformation, that is, those in which hTERT (telomerase) has been ectopically expressed, do not die due to the action of the drug, but become senescent (drug-induced sencescence). Disruption of the pathways controlled by the tumour suppressors p53 and pRb is necessary and sufficient to set the conditions for the acquisition of drug resistance. The diagram is based on data from Yagüe *et al* (2007).

The use of drug-resistant derivatives from human and other mammalian cell lines has been of paramount importance for the unravelling of many of the mechanisms of cancer drug resistance highlighted above. However, they have proved less successful in identifying the ultimate upstream regulators controlling these events, and as such, how drug resistance arises is still unresolved. Most cellular models of drug resistance have been developed from transformed cell lines isolated from patients at a late stage in cancer progression and whose tumours already exhibit a plethora of karyotypic and physiologic abnormalities. Recently, several groups have shown that it is possible to transform primary human cells into fully tumorigenic cells by altering a small number of defined pathways *ex vivo*. This cellular model of tumorigenesis (Figure 1A) was first described by Hahn *et al* (1999) in human BJ fibroblasts and embryonic kidney epithelial cells by expressing the catalytic subunit of telomerase (to avoid replicative senescence), SV40 large T-antigen (which binds and inactivates the tumour suppressors p53 and pRb controlling the DNA repair and G1 cell cycle checkpoints, respectively) and small t-antigen (which binds and inactivates PP2A, a serine/threonine phosphatase involved in several signalling pathways) and oncogenic ras. Primary human epithelial cells from the mammary gland, prostate, ovary, trachea and bronchia have now been transformed by introducing these or similar sets of genes (Boehm and Hahn, 2005). Although somatic p53 missense mutations are found in approximately 50% of human cancers, the p53 pathway can also be inactivated in wild-type p53-carrying tumours by p53 destabilisation via indirect mechanisms such as *MDM2/MDMX* amplification. In addition, most wild-type p53 types of cancer harbour alternative genetic alterations such as mutations in *APC* in colon cancer, *BRCA1* and *BRCA2* in breast cancer, and *B-RAF* in melanoma. As the p53 network is closely linked to many other cellular pathways, it is likely that defects in any of these pathways could alter p53 function (Soussi and Wiman, 2007).

The development of these minimally transformed cells has, for the first time, allowed us to ask whether the capacity to develop drug resistance arises before or after tumorigenic transformation, and what is the minimum number of altered pathways required to permit this event. In a series of progressively transformed embryonic skin fibroblasts, it has been found that the minimum number of genetic transformations necessary for a primary cell to become drug resistant, in addition to hTERT expression, is inactivation of the pathways controlled by p53 and pRb (Yagüe *et al*, 2007), confirming the pivotal roles of p53 and pRb in deciding cell fate after drug treatment: senescence, apoptosis or drug resistance (Dimri, 2005). Thus, in this cell model, the ability to acquire drug resistance is not, as previously supposed, a late event in tumorigenesis resulting from gross genetic instability, but is intrinsic to the early steps in the tumorigenic pathway necessary for transformation and can arise earlier than the full malignant transformation (Figure 1B).

These findings with minimally transformed fibroblasts need to be confirmed and extended to other epithelial cancer cell models and to drugs with different modes of action to generalise their relevance. However, they have opened the possibility to analyse the ultimate controllers in the development of drug resistance.

## Cancer stem cells and drug resistance

The cancer stem cell (CSC) hypothesis states that many, if not all, cancers contain a minority population of transformed self-renewing stem cells. These CSCs are responsible for sustaining the tumour as well as giving rise to proliferating but progressively differentiating cells constituting the tumour mass (Burkert *et al*, 2006; Li and Neaves, 2006). Cancer stem cells retain the essential property of self-protection through the activity of multiple drug resistance transporters such as ABCB1 (P-glycoprotein) and/or ABCG2 (Breast Cancer Resistance Protein-1, BCRP1). The latter is responsible for the side-population (SP) phenotype detected in both normal and acute myeologenous leukaemia (AML) haematopoietic stem cells (Wulf *et al*, 2001). As *Mdr1a/1b*^−/−^ (P-glycoprotein-deficient) mice are able to display a normal SP phenotype that disappears when *Abcg2* is knocked down (Zhou *et al*, 2002), expression of ABCG2 and Hoechst 33342 efflux are two of the best markers of these cells. To date, the existence of CSCs has been demonstrated in AML and CML, in brain and gastrointestinal tumours, and in lung and breast cancer (de Jonge-Peeters *et al*, 2007).

A connection between CSCs and drug resistance is thought to exist due to the expression of many of the membrane transporters. In fact, the whole drug resistance concept has been revised incorporating the CSC paradigm (Dean *et al*, 2005; Donnenberg and Donnenberg, 2005). According to the acquired resistance stem-cell model, CSCs, which express drug transporters, are present in the original tumour mass and survive chemotherapy, whereas the committed but variably differentiated cells are killed. These cells reform a heterogeneous drug resistant tumour composed of CSCs and a committed but variably differentiated offspring. In addition, mutation in the surviving CSCs can arise expanding the drug-resistant phenotype. However, the CSC hypothesis does not account for resistance that develops in certain cancers following chemotherapy in which all cancer cells (not just stem cells) become resistant. Such intrinsic drug resistance is in many cases, such as colon and liver cancer, due to the function of ABC transporters, which are already highly expressed in the healthy tissues.

## Drug resistance in the clinic and its reversal

Cell culture systems and animal models have been pivotal in defining the main molecular and cellular mechanisms responsible for the drug-resistance phenotype. With them it has been relatively straight forward to demonstrate that a particular molecule (i.e. P-glycoprotein or p53) is the effector of drug resistance, due to the ease of performing knockout or ectopic expression experiments. However, the situation in the clinic is far more complicated, not only because of the lack of sensitivity and the absence of appropriate detection techniques from clinical samples, but also because the association of a particular drug-resistance effector does not necessarily correlate with an alteration in the chemotherapy response (Cimoli *et al*, 2004). Despite this, a negative correlation has been unequivocally demonstrated between P-glycoprotein expression and chemotherapy response in AML (Gottesman *et al*, 2002) and breast cancer (Clarke *et al*, 2005).

Since the discovery of P-glycoprotein in the early 1980s, most agents tested for the reversal of multidrug resistance in the clinic have aimed at inhibiting P-glycoprotein function. The first generation of P-glycoprotein inhibitors included verapamil, quinine and cyclosporine, which were already approved for other medical purposes. Although these compounds proved to be ineffective or toxic at the doses required to attenuate P-glycoprotein function, some clinical trials indicated that modulation of P-glycoprotein function could be achieved (Gottesman *et al*, 2002). This encouraged the development of a second-generation of modulators, such as the cyclosporine analogue PSC-833 (Valspodar), aimed at avoiding the toxic side effects seen in the first generation. However, the development of second-generation inhibitors has now been discontinued, mainly due to their limited success in clinical trials (PSC-833 induced pharmacological interactions that limited drug clearance and metabolism of the chemotherapeutic agent, thereby elevating plasma concentrations beyond acceptable toxicity). Third-generation inhibitors have been designed for low pharmacokinetic interaction, and inhibition of cytochrome *P*450 3A has been avoided with compounds such as laniquidar (R101933), oc144-093 (ONT-093), zosuquidar (LY335979), elacridar (GF-120918) and tariquidar (XR9576). A further generation of inhibitors acts on a broader range of ABC transporters. These include biricodar (VX-710) and GF-120918, which modulate not only P-glycoprotein but also MRP1 and ABCG2, respectively. Most clinical trial end points have not been analysed yet; for an extended discussion of P-glycoprotein inhibitors in the clinic see Szakacs *et al* (2006).

There are many different possible reasons for the failure of phase III clinical trial targeting P-glycoprotein. These include multifactorial mechanisms of resistance, toxicity of the inhibitors and unfavourable pharmacological interactions, as well as a poor clinical trial design. The latter is exemplified by the phase III clinical trial using tariquidar as an adjunctive treatment in combination with first-line chemotherapy for patients with non-small cell lung carcinoma, in which there is no strong evidence to suggest that in this type of cancer, P-glycoprotein is expressed to a significant extent (Szakacs *et al*, 2006). Even AML patients, in which P-glycoprotein expression affects the outcome of chemotherapy, are not routinely phenotyped, and current efforts to develop simple, intercentre reproducible protocols to detect P-glycoprotein in AML blasts have been developed (Pallis *et al*, 2005). In such a way, trial organisers can have the choice of whether to give P-glycoprotein modulators to an unsorted cohort or to P-glycoprotein-positive patients only.

Other alternative approaches to target P-glycoprotein-mediated drug resistance could involve the development of agents to interfere with any one of the regulatory steps in P-glycoprotein expression: transcription, mRNA turnover, translation, protein processing and turnover. Of these, the only one under trial in soft tissue sarcoma (currently phase II) is ecteinascidin 743, a natural product isolated from the marine organism *Ecteinascidia turbinate*. Ecteinascidin 743 interferes with the activation of *ABCB1 via* the stress-responsive enhanceosome complex (Le Cesne *et al*, 2005).

Genome-wide expression profiling has been used to study drug resistance, and the foreseeable complexity of their mechanisms has been revealed. In addition to the corroboration of traditionally associated genes, these studies have given new insight into the regulatory networks controlling drug resistance (Turton *et al*, 2001). The most interesting application of the novel genomic technologies has been in the clinical arena, where molecular signatures have been used not only to characterise neoplastic transformation (Sotiriou *et al*, 2003) and resistant tumours (Jansen *et al*, 2005) but also, and most importantly, to predict the outcome of chemotherapy (Alaoui-Jamali *et al*, 2004).Whether these novel technologies will gain acceptance in the routine diagnosis of cancer will depend greatly on whether their current costs can be reduced (Sotiriou and Piccart, 2007).

## Conclusions and perspectives

Many years have passed since the first description of cancer as an evolutionary process (Nowell, 1976). With the establishment of the CSC paradigm, new insights into the relative high frequency of cancer in humans and the pitfalls of many cancer treatments have been put forward based on Darwinian selection (Greaves, 2007). Although there are many differences between neoplastic and organismal evolution, the lack of cellular controls to maintain genomic stability, telomere length, repair of DNA damage or cell cycle regulation discussed above set up the conditions for genetic diversity as a source of clonal evolution. Treatment with chemotherapeutic agents promotes an evolutionary arms race exemplified by the resistance to imatinib and the generation of novel derivatives in CML. Studies using cell model systems have shown that chemotherapy resistance is intrinsic to the tumorigenesis process and can even arise before malignant transformation. Thus, possible solutions lie in the development of novel approaches, based mainly on immunological and gene therapy techniques, aiming to at least partially restore some of the normal cellular controls, although cancer gene therapy is still in its infancy. Whether these or other still-to-come therapies will allow us to say in future years, paraphrasing the Borg from Star Trek, ‘Resistance is futile’ is difficult to foresee, but as Captain Jean-Luc Picard stated ‘Things are only impossible until they're not’.
